# Humoral responses in *Rhodnius prolixus*: bacterial feeding induces differential patterns of antibacterial activity and enhances mRNA levels of antimicrobial peptides in the midgut

**DOI:** 10.1186/1756-3305-7-232

**Published:** 2014-05-20

**Authors:** Cecilia Stahl Vieira, Peter J Waniek, Débora P Mattos, Daniele P Castro, Cícero B Mello, Norman A Ratcliffe, Eloi S Garcia, Patrícia Azambuja

**Affiliations:** 1 Laboratório de Bioquímica e Fisiologia de Insetos, Instituto Oswaldo Cruz, Fundação Oswaldo Cruz (Fiocruz), Rio de Janeiro, Rio de Janeiro, Brazil; 2 Laboratório de Biologia de Insetos, Departamento de Biologia Geral, Instituto de Biologia, Universidade Federal Fluminense (UFF) Niterói, Rio de Janeiro, Brazil; 3 College of Science, Swansea University, Swansea, Wales, UK; 4 Departamento de Entomologia Molecular, Instituto Nacional de Entomologia Molecular (INCT-EM), Rio de Janeiro, Rio de Janeiro, Brazil

**Keywords:** *Rhodnius prolixus*, Antimicrobial peptides, Bacteria, mRNA modulation

## Abstract

**Background:**

The triatomine, *Rhodnius prolixus,* is a major vector of *Trypanosoma cruzi*, the causative agent of Chagas disease in Latin America. It has a strictly blood-sucking habit in all life stages, ingesting large amounts of blood from vertebrate hosts from which it can acquire pathogenic microorganisms. In this context, the production of antimicrobial peptides (AMPs) in the midgut of the insect is vital to control possible infection, and to maintain the microbiota already present in the digestive tract.

**Methods:**

In the present work, we studied the antimicrobial activity of the *Rhodnius prolixus* midgut *in vitro* against the Gram-negative and Gram-positive bacteria *Escherichia coli* and *Staphylococcus aureus*, respectively. We also analysed the abundance of mRNAs encoding for defensins, prolixicin and lysozymes in the midgut of insects orally infected by these bacteria at 1 and 7 days after feeding.

**Results:**

Our results showed that the anterior midgut contents contain a higher inducible antibacterial activity than those of the posterior midgut. We observed that the main AMP encoding mRNAs in the anterior midgut, 7 days after a blood meal, were for lysozyme A, B, defensin C and prolixicin while in the posterior midgut lysozyme B and prolixicin transcripts predominated.

**Conclusion:**

Our findings suggest that *R. prolixus* modulates AMP gene expression upon ingestion of bacteria with patterns that are distinct and dependent upon the species of bacteria responsible for infection.

## Background

Although insect immunity has been studied since the first half of the 20th century [1-3], the mechanisms involved have yet to be fully elucidated. The immune system in insects, unlike vertebrates, lacks the classical response to pathogens mediated by memory cells and immunoglobulin, but relies solely on an extremely efficient innate immune response [4]. This efficiency is probably one reason insects are the most abundant animal group, well adapted to many ecotopes [5]. Insect immunity includes the synchronized activation of cellular and humoral factors, such as the formation of microaggregates, phagocytosis and encapsulation by haemocytes, as well as the formation of reactive intermediates of oxygen and nitrogen, the prophenoloxidase system and antimicrobial peptides (AMPs) [6,7].

One of the major components of insect immunity is the synthesis of AMPs. Insect AMPs are usually cationic, amphipathic, often composed of 12–50 amino acid residues and have a broad activity spectrum [8]. The gene expression of AMPs occurs principally in the fat body, haemocytes and digestive tract epithelia, and the peptides are secreted into the haemolymph or midgut lumen [9,10]. AMP production is triggered by activation of different immune signalling pathways including Toll, Imd and Jak/STAT after recognition of non-self molecules known as the pathogen associated molecular patterns (PAMPs) [5,11].

Relatively few studies focus on the importance of the immune system in the midgut of insects, which is one of the most vulnerable tissues since it is always in contact with a variety of microorganisms [12]. Haematophagous insects, such as *Rhodnius prolixus,* ingest large amounts of blood from vertebrate hosts, often containing pathogenic microorganisms. The production of AMPs in the insect gut is therefore vital to protect against infection and to maintain homeostasis of the intestinal microbiota. The mutualistic microbiota of insects not only supplies essential nutrients but also aids digestion and the control of pathogenic microorganisms by modulating the immune responses [13,14]. Moreover, several studies have shown the importance of the microbiota in regulating insect genes involved in maintaining homeostasis of the gut [15-21].

*R. prolixus* is an important triatomine vector of *Trypanosoma cruzi*, the etiologic agent of Chagas disease in Latin America [22-24]. In the insect vector, *T. cruzi* remains exclusively inside the *R. prolixus* gut where, in order to survive, the parasite counteracts various host defence factors, including the AMPs [12]. Evidence indicates that in some insect vectors AMPs may be able to control parasite development [25-30]. Therefore, the study of AMPs present in the digestive tract of insects may have potential to provide new targets for control strategies.

Antimicrobial peptides are encountered in numerous organisms and are diverse even among closely related species [8]. In *R. prolixus,* six different AMPs have been identified, namely, defensin A, B and C, prolixicin and lysozymes A and B [31]. Each AMP has potential activity against a range of microorganisms. Lysozymes possess high activity against Gram-positive bacteria, by hydrolysing the 1,4-β-linkage between N-acetylmuramic acid and N-acetylglucosamine of the cell wall peptidoglycans [32,33]. Defensins are cysteine-rich peptides and are also known for their action against Gram-positive bacteria [27,34-36]. In contrast, prolixicin has high activity against Gram-negative *Escherichia coli*[37].

Despite the presence of these different AMPs in *R. prolixus*, the relative dynamics of their induction upon exposure to different species of bacteria is poorly understood. Thus, in the present study, using fifth instar nymphs of *R. prolixus*, the antimicrobial activities of the midgut *in vitro* against *Staphylococcus aureus* and *E. coli* have been investigated. We also analysed the relative abundance of mRNAs encoding AMPs in the midgut of insects fed with either *S. aureus* or *E. coli* at different days after an infected blood meal to test the hypothesis that each type of bacterium triggers a distinct immune response.

## Methods

### Ethics statement

For all experiments, *R. prolixus* were maintained in controlled environmental conditions and fed with defibrinated rabbit blood provided by the Laboratory Animals Creation Centre (Cecal). For feeding insects, an artificial apparatus was used, similar to that described previously [38] according to the Ethical Principles in Animal Experimentation approved by the Ethics Committee in Animal Experimentation (CEUA/FIOCRUZ, under the protocol number L-0061/08). The protocol was developed by CONCEA/MCT (http://www.cobea.org.br/), which is associated with the American Association for Animal Science (AAAS), the Federation of European Laboratory Animal Science Associations (FELASA), the International Council for Animal Science (ICLAS) and the Association for Assessment and Accreditation of Laboratory Animal Care International (AAALAC).

### Bacteria

*S. aureus* 9518 and *E. coli* K12 4401 were purchased from the National Collections of Industrial and Marine Bacteria (NCIMB), Aberdeen, UK. Bacteria were maintained frozen at −70°C in tryptone agar and 10% glycerol. For all experimental procedures, bacteria were grown with shaking (90 revolutions per minute) in 20 ml of tryptone soy broth (TSB) for 17 h at 30°C, and then 10 ml of fresh TSB were inoculated with 100 μl of the respective bacterial culture and incubated for a further 4 h under the same conditions. The bacteria were then washed in phosphate buffered saline - PBS (0.01 M phosphate buffer, 2.7 mM potassium chloride and 0.137 M sodium chloride, pH 7.4) and diluted in TSB to a final concentration of 1 × 10^4^ cells/ml.

### Insect treatment

Fifth-instar *R. prolixus* nymphs were obtained from a colony reared and maintained in Laboratório de Bioquímica e Fisiologia de Insetos IOC/FIOCRUZ at a relative humidity of 50–60% and at 27°C. The insects were randomly chosen and then fed with defibrinated rabbit blood through a membrane feeding apparatus [38]. Three groups of insects were fed as follows: blood only (control), blood containing *E. coli* or blood containing *S. aureus*. The final concentration of bacteria in the blood was 10^4^/ml.

To compare the effects of whole normal plasma on the insect’s antibacterial activity, insects were fed with blood after heat-inactivation of the plasma. The blood was centrifuged at 1.890 × g for 10 min at 4°C, and the supernatant (plasma) was collected and incubated for 30 min at 55°C. After inactivation, the plasma was added back to the erythrocytes and fed to the insects. In the same experiment, a group of insects was fed with normal plasma in the blood (control).

### Midgut sample preparations and antibacterial assays

For midgut sample preparations, starved or full engorged fifth-instar nymphs of *R. prolixus* were used at different days after feeding (DAF). The cuticle of the insects was cut laterally, to remove and separate the enlarged anterior midgut (stomach) from the narrow posterior midgut (intestine). The anterior midgut was separated into contents and wall for the antibacterial assays. Additionally, the antibacterial activity of the intestine was tested. All midgut preparations were collected in 1.5 ml reaction tubes always using pools of 3 insect midgut compartments diluted in 200 μl Milli-Q water, homogenized, centrifuged at 10,000 × g for 10 min at 4°C and finally sterilized by Millipore PVDF membrane filtration. Afterwards, the pools of 3 anterior midgut contents were diluted ten times in sterile water and stored at −20°C until use.

Antibacterial activity was assessed by turbidometric assays (TB) previously adapted by Castro *et al.,* 2012 [39,40]. For midgut TB assays, *S. aureus* or *E. coli,* grown as described above, were washed in PBS and diluted in TSB to a final concentration of 10^4^ cells/ml. Subsequently, 10 μl of *E. coli* or *S. aureus* bacterial suspensions were incubated in each well of a sterile flat bottom 96-well microtiter plate (Nunc, Fisher Scientific, Leicestershire, UK) with 45 μl of sample (anterior midgut content, anterior midgut wall or posterior midgut) plus 5 μl of peptone 10%, to a final concentration of 1% peptone, at 37°C for 19 h. The optical densities were measured at 550 nm (OD_550_) at hourly intervals using a Spectra Max 190 Plate Reader (Molecular Devices, Sunnyvale, California, USA). Control wells, run without midgut samples, contained 10 μl of bacteria in 1% peptone in Milli-Q water. The antibiotic ampicillin (80 μg/ml) was included in each experiment as a positive control.

All data points were subsequently blanked against time zero to account for the opacity of the midgut samples. The midgut samples were also incubated in the plate without bacteria to observe the change in sample colour after 19 h and the readings obtained were subtracted from the samples incubated with bacteria to ensure that the difference in readings were related to antibacterial activity. Then, the readings for the bacteria, *E. coli* or *S. aureus,* were subtracted from all sample readings to obtain the antibacterial activity value. All experiments were carried out in triplicate (9 pools of 3 insects, n = 27 insects). In addition, to find out how the sample dilutions affect antibacterial activity, different concentrations of the anterior midgut contents were tested against both bacteria. The anterior midgut contents of control insects at 7 DAF without dilution gave absorbance readings above the range of the standard curve and therefore in all TB assays samples were diluted 10 times which corresponded to 14.7 μg protein/μl of protein. The posterior midgut samples of control insects at 7 DAF used for TB assays contained 0.8 μg protein/μl of sample. All protein testing of midgut samples used a protein assay kit (BCA* Protein Assay Reagent, Pierce, USA) with bovine serum albumin (BSA) standards. Additionally, the differences in protein concentrations of each midgut preparation analysed in these assays were considered and are discussed below.

Concurrent with the TB assays, the anterior and posterior midgut samples (45 μl) were also incubated with 10 μl of *E. coli* or *S. aureus* (1 × 10^4^ cells/ml) and 5 μl of peptone 10% at 37°C. At different times during incubation, samples were plated onto BHI-agar to compare the bacterial growth, by counting colony forming units (CFU), with the readings in the TB assays. Ampicillin (80 μg/ml) was incubated with both bacteria and plated on BHI-agar as a positive control of bacterial growth inhibition. The culture medium (TSB) used in the sample dilutions was also plated out as a control.

The thermal stability of the anterior midgut contents was analysed by heating the samples at 100°C for 60 min. The susceptibility of the anterior midgut contents to protease digestion was tested by pre-incubation with bovine pancreas trypsin (Sigma-Aldrich) at a final concentration of 2500 Uml^−1^ for 24 h at 37°C [40]. Samples were then centrifuged at 10,000 × g for 5 min and the supernatants assayed for antibacterial activity. Tests showed that trypsin had no adverse effects on bacterial growth and for this reason was not inhibited in the sample prior to TB assay.

### Analysis of AMPs mRNA abundance by reverse transcription (RT) PCR

Steady state levels of mRNA encoding peptides involved in the innate immunity of *R. prolixus* were tested by reverse transcription (RT) PCR. Before dissection, insects were immersed in water at 55°C for 15 sec to release haemocytes from tissues [41]. From fifth instar nymphs (n = 10), unfed (15 days after ecdysis), 1 and 7 DAF (infective and non-infective), the anterior and posterior midgut walls were dissected and stored at −70°C. Total RNA was extracted using a NucleoSpin® RNA II Kit (Macherey-Nagel, Düren, Germany) following the manufacturer’s instructions and subsequently measured by a NanoDrop 2000 Spectrophotometer (Thermo Scientific, Waltham, MA, USA). Synthesis of cDNA was carried out with a First-Strand cDNA Synthesis Kit (GE Healthcare, Buckinghamshire, UK) following the manufacturer’s protocol using either 1.25 or 2.5 μg of total RNA. *R. prolixus* primers were designed from previously published defensin A, B and C, lysozyme A and B, prolixicin and β-actin (internal control, GenBank accession number ACPB02032143) encoding cDNA sequences as listed in Table 1[31,37,42-44]. All defensins and the prolixicin encoding genes possess an intron and could therefore also be used as an internal control for contamination with genomic DNA.

**Table 1:** List of primers used in the present study

**Gene/name**	**Sequence 5’-3’**	**Tm (°C)**	**Amplicon length**
RPDEFAF	GAATACTCCACTCAACCGCAAC	62.7	
RPDEFAR	TAGTTCCTTTACATCGGCCA	58.4	295 bp
RPDEFBF	CAGTACCTAGGATATTCCACTCAAC	62.9	
RPDEFBR	TAGTTCCTTTACAATGGCCG	58.4	304 bp
RPDEFCF	CAGTACAGTCCTAATACCTAGCC	62.8	
RPDEFCR	CAGTTCCTACGCAACGGCCT	64.5	300 bp
RPLYS1F	TTCTTACTGGCTATTTTCGCC	58.7	
RPLYS1R	CGACCTCTGCAATGGTACTG	62.4	377 bp
RPLYS2F	CTAGTTTTAACACTATTGCTGCTG	59.4	
RPLYS2R	GCCCTTACATTTCTTGATCC	58.4	378 bp
RPPROLF	CTATAACGAGTGAACTATAAGACAA	50.0	
RPPROLR	GTGTTTAATGGCGGTAACAAATTAC	53.2	406 bp
RPACTF	CACGAGGCTGTATACAATTCCA	60.8	
RPACTR	GTAGCTGTTTAGAAGCATTTGCG	61.0	314 bp

PCRs were performed using Illustra Taq DNA Polymerase (GE Healthcare, Buckinghamshire, UK) at the following conditions: initial denaturation at 94°C for 5 min; cycling step at 94°C for 25 sec, 54°C for 25 sec, 72°C for 30 sec and a final elongation step at 72°C of 7 min. The amplification of prolixicin was conducted at an annealing temperature of 48°C. The number of cycles (25 and 30) was experimentally optimized with the gene encoding actin to eliminate signal saturation [45]. For verification of primer specificity, amplicons of all genes were excised from agarose gels, purified and sequenced in both directions by Plataforma Genômica − Sequenciamento de DNA/PDTIS-FIOCRUZ, Rio de Janeiro, Brazil. PCRs were carried out three times under the same conditions using technical replicates. As negative controls, PCR reactions were carried out without a template. All nucleic acid experiments were performed on a Veriti 96-Well Fast Thermal Cycler (Applied Biosystems, Carlsbad, CA, USA). Amplification products (5 μl) were separated on an ethidium bromide stained 2% agarose gel and documented with a Gel Doc™ XR + System (Bio-Rad, Hercules, CA, USA). Band intensity was measured with the ImageJ program (version 1.47q). Means and standard deviations of the different samples were calculated.

### Statistical analyses

The results were analysed with GraphPad Prism 5 using two way ANOVA or one way ANOVA or unpaired T tests, depending on the data distribution and number of treatments. Data are reported as mean ± standard deviation (SD). Differences among groups were considered not statistically significant when p > 0.05. Probability levels are specified in the text and Figure legends.

## Results

### Midgut antimicrobial activity

**Figure 1:**
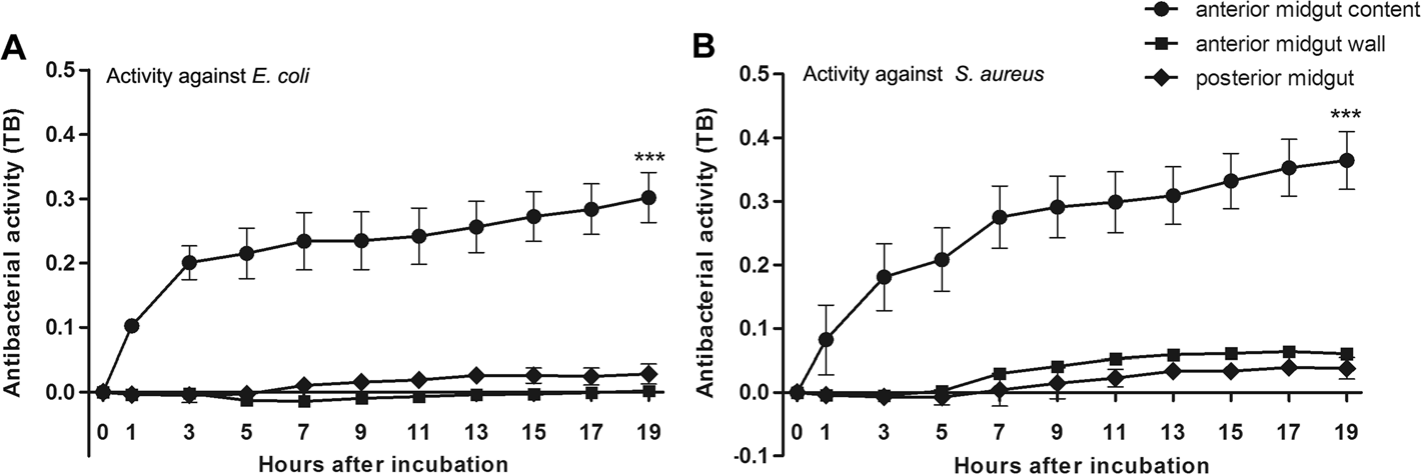
**Antibacterial activity of the anterior and posterior midgut of *Rhodnius prolixus *7 days after feeding*. *A** – Activity of anterior (contents and wall) and posterior midgut samples against *E. coli*. **B** – Activity of anterior (contents and wall) and posterior midgut samples against *S. aureus*. Antibacterial activity measured by turbidometric assay (TB) (OD_550_ nm) with readings from 0 to 19 hour in plate assay. Treatments: ● bacteria incubated with anterior midgut contents; ■ bacteria incubated with anterior midgut wall; ♦ bacteria incubated with posterior midgut. Values represent the means ± SD of 9 pools using 3 insects each (n = 27) in triplicate wells. Asterisks relates to significant differences (***p < 0.001) obtained by a two way ANOVA.

In the present study, the antimicrobial activity of *R. prolixus* midgut was assessed against two bacterial species, *E. coli* and *S. aureus*. To determine in which midgut compartment the antibacterial activity are present, we tested separately the anterior midgut wall and contents as well as total posterior midgut using the TB assay (Figure 1). Results showed that the anterior midgut contents had a significantly higher activity than the anterior midgut wall and posterior midgut against both bacterial species (Figure 1; p < 0.001). A comparison between the anterior midgut contents and posterior midgut was also made using BHI agar plates incubating the samples with *E. coli* and *S. aureus*. In the anterior midgut contents, no bacteria grew after 19 h incubation in contrast to the rapid growth of the bacteria alone controls (Additional file 1; p < 0.001). In contrast, incubation with the posterior midgut samples resulted in numerous bacteria colony forming units (CFU) after 19 h (Additional file 1; p < 0.001). These results confirm those from the TB assay above.

Analysis was also undertaken to determine if any antibacterial activity recorded was related to the complement system of the rabbit blood. Comparison of the antibacterial activity of anterior and posterior midgut samples from insects fed on blood containing whole native plasma with those fed on heat- inactivated plasma revealed no differences in activity against *E. coli* or *S. aureus* (Additional file 2).

**Figure 2:**
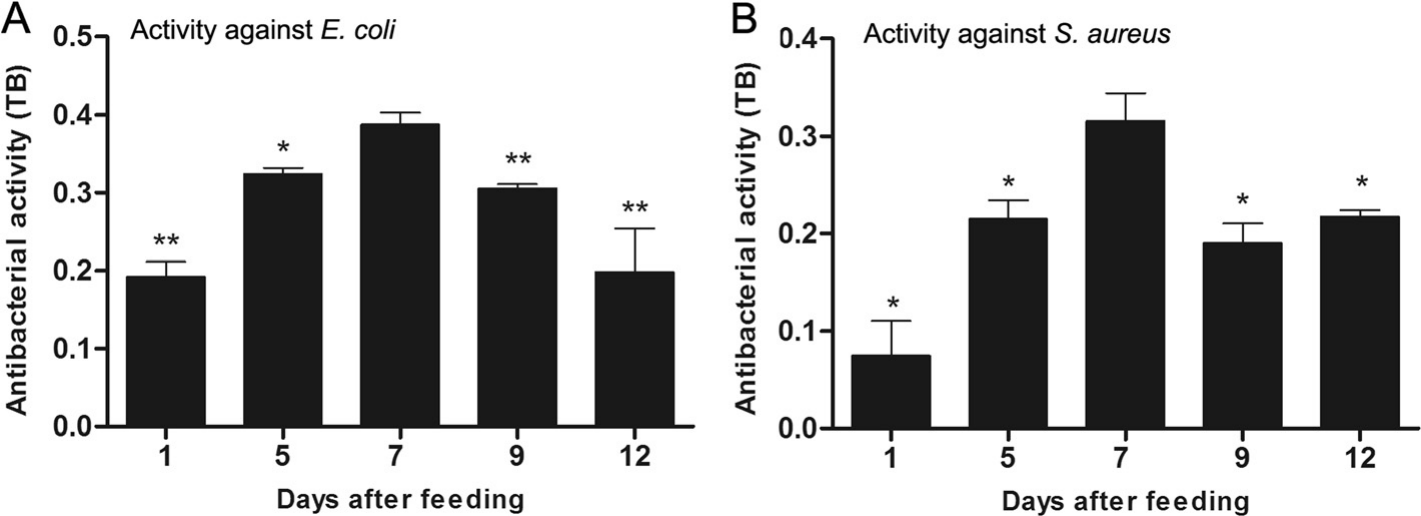
**Antibacterial activity of the anterior midgut contents of *Rhodnius prolixus *on different days after feeding. A**- Activity of anterior midgut contents against *E. coli*. **B**- Activity of anterior midgut contents against *S. aureus*. Antibacterial activity detected by turbidometric assay (TB) (OD_550_ nm) after 19 h incubation of anterior midgut content samples with different bacteria. Values represent the means ± SD of 9 pools using 3 insects each (n = 27) in triplicate wells. Asterisks relate to significant differences (*p < 0.05, **p < 0.01, ***p < 0.001) obtained after data were compared to day seven using one way ANOVA and Mann Whitney tests.

In order to analyse the dynamics of antibacterial activity in *R. prolixus*, the anterior midgut contents were tested against *E. coli* or *S. aureus* at different days after feeding (DAF). The results showed that at 7 DAF, the activity against *E. coli* was significantly higher than 5 DAF (p < 0.05), as well as 1, 9 and 12 DAF (p < 0.01) (Figure 2A). The activity of the anterior midgut contents against *S. aureus* was also highest at 7 DAF which was significantly higher (p < 0.05) than all the other DAF (Figure 2B).

The antibacterial activity of the anterior midgut contents was also tested for thermal stability and susceptibility to trypsin digestion. All antibacterial activities against *E. coli* and *S. aureus* were significantly reduced after trypsin and boiling treatments compared with the untreated controls (Additional file 3A; p < 0.01 and p < 0.05, respectively). The activities against *S. aureus* were also significantly reduced with these treatments (Additional file 3B; p < 0.001).

### Transcription of AMPs in insects

**Figure 3:**
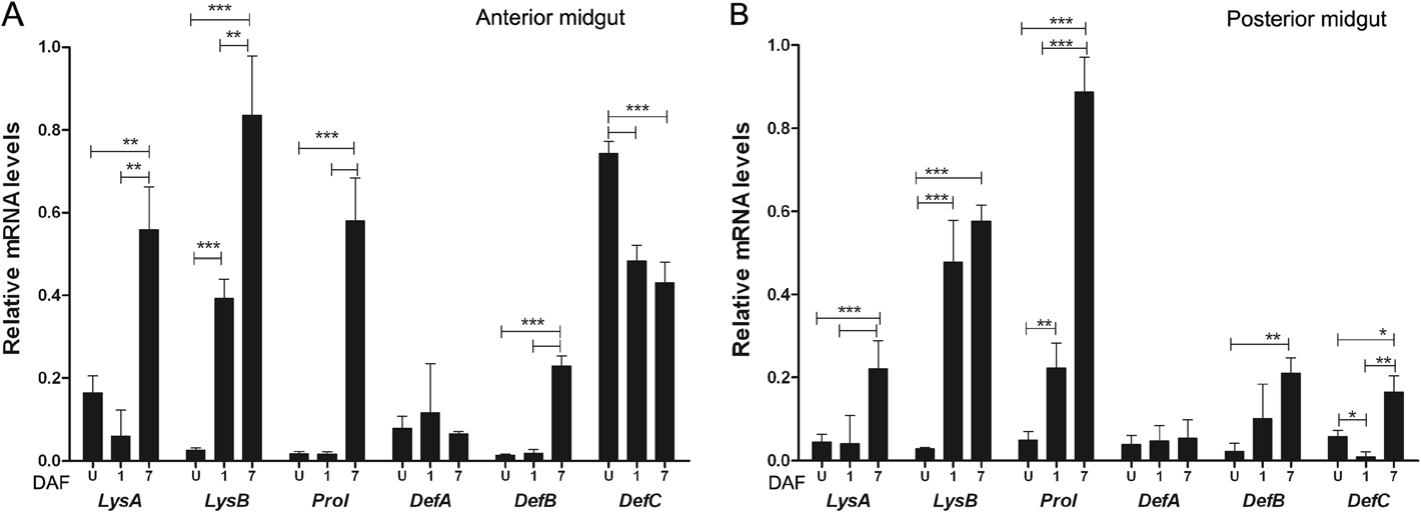
**Relative transcript abundance of antimicrobial peptides and lysozymes encoding mRNA in *Rhodnius prolixus *midgut wall.** Anterior and posterior midgut samples collected before feeding (unfed)*,* 1 and 7 days after a blood meal. **A**- Relative mRNA levels in anterior midgut. **B**- Relative mRNA levels in posterior midgut. DAF – days after feeding. U – unfed insects. Error bars represent SD of three independent experiments. Asterisks relates to significant differences (*p < 0.05, **p < 0.01, ***p < 0.001) obtained after data analyses using one way ANOVA and unpaired t tests.

In order to categorize antibacterial activity in the digestive tract of *R. prolixus*, the gene expression profiles of AMPs in the anterior midgut and posterior midgut walls of unfed insects and insects 1 or 7DAF were studied. The relative abundance of transcripts for lysozyme A (*LysA*), lysozyme B (*LysB*), prolixicin (*Prol*), defensins A (*DefA*), B (*DefB*) and C (*DefC*) was quantified (Figure 3). In general, the AMP transcript abundance was highest at 7 DAF in both tissues, but the expression pattern over time and tissue was not the same for all AMPs analysed (Figure 3A and 3B). At 1 DAF, the abundance of transcripts of *LysB* increased approximately 15 fold in the anterior and posterior midguts, while *Prol* transcripts increased 5 fold in the posterior midgut, in comparison to unfed insects. Interesting, *DefC* abundance was significantly higher in anterior midgut samples of unfed insects (p < 0.001), and decreased at 1 and 7 DAF (Figure 3A). Comparing the transcripts between tissues 7 DAF, the anterior midgut showed a significantly higher abundance of *LysA*, *LysB* and *DefC* than the posterior midgut (Figure 3A). Additionally, only the abundance of *Prol* transcripts was significantly higher in posterior midgut than anterior midgut (Figure 3B).

### Antibacterial activity and transcription of AMPs in bacteria fed insects

**Figure 4:**
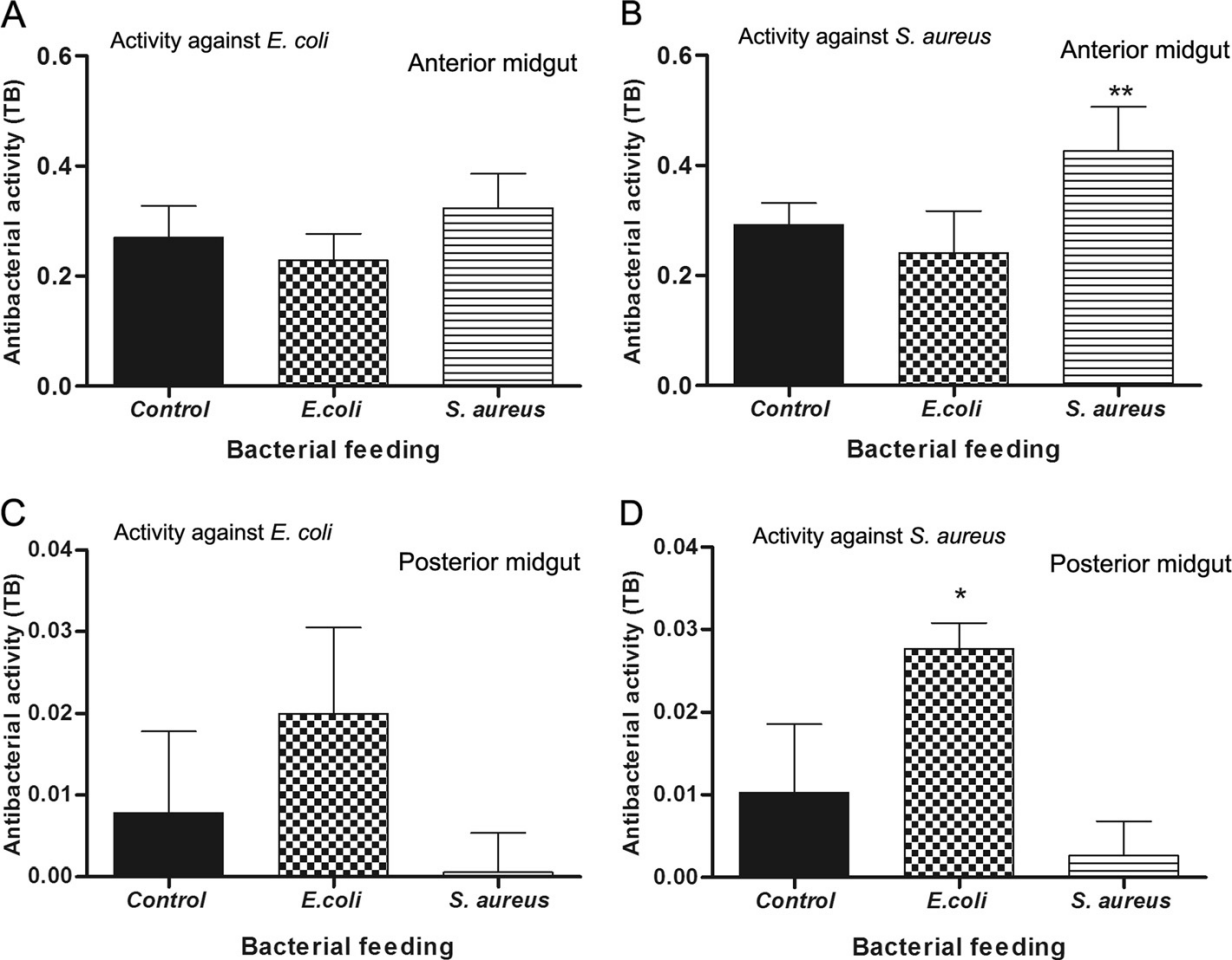
**Antibacterial activity in *Rhodnius prolixus *midgut fed with blood containing *E. coli *or *S. aureus*.** Anterior midgut contents and posterior midgut collected 7 days after feeding were tested against *E. coli* and *S. aureus*. **A-** Antibacterial activity of anterior midgut contents after feeding with *E. coli*, *S. aureus* or blood alone against *E. coli*. **B**- Antibacterial activity of anterior midgut contents after feeding with *E. coli*, *S. aureus* or blood alone against *S. aureus*. **C-** Antibacterial activity of posterior midgut after feeding with *E. coli*, *S. aureus* or blood alone against *E. coli*. **D**- Antibacterial activity of posterior midgut after feeding with *E. coli*, *S. aureus* or blood alone against *S. aureus*. Black column - antibacterial activity of control insects fed on blood alone; grid column - antibacterial activity of insects fed with blood containing *E. coli*; striped column - antibacterial activity of insects fed with blood containing *S. aureus*. Antibacterial activity measured by turbidometric assay (TB) (OD_550_ nm) after 19 h incubation of midgut samples with different bacteria. Values represent the means ± SE of three replicates. Asterisks relates to significant differences (*p < 0.05, **p < 0.01, ***p < 0.001) in comparison to control obtained after data analyses using one way ANOVA and Mann Whitney tests.

*R. prolixus* were infected separately with Gram-positive and Gram-negative bacteria to test whether different bacteria trigger a distinct immune response, altering the antibacterial activity and the gene expression of AMPs. The antibacterial activities recorded were compared to control insects fed on blood without bacteria. Feeding the insects with blood containing *E. coli* failed to significantly alter the immune response of the anterior midgut contents tested against either *E. coli* or *S. aureus* (Figure 4A and 4B). In contrast insects fed with *S. aureus* recorded significantly increased antibacterial activity of the anterior midgut contents against *S. aureus* (Figure 4B; p < 0.01) but not *E. coli*. As with the anterior midgut contents, the oral infection with either bacterium failed to significantly change the antibacterial activities of the posterior midgut samples against *E. coli* (Figure 4C), although an increase in posterior midgut antibacterial activity was only observed afterwards when the insects were infected with *E. coli* and then tested against *S. aureus* (Figure 4D; p < 0.05).

**Figure 5:**
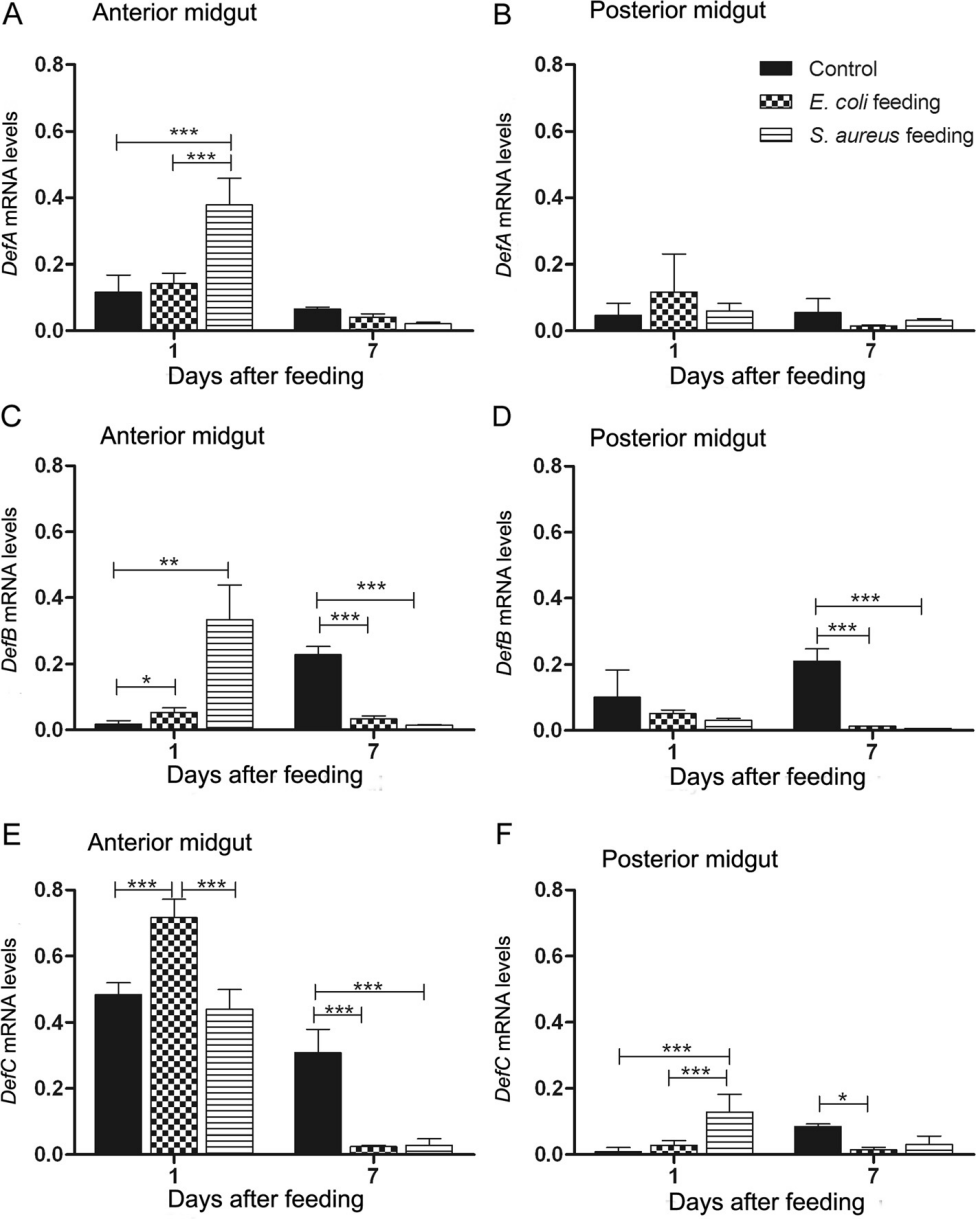
**Relative transcript abundance of defensins encoding mRNA in *Rhodnius prolixus *after bacterial feeding.** Anterior and posterior midgut samples collected 1 and 7 days after blood meal. **A, C, E**: anterior midgut relative mRNA levels. **B, D, F**: posterior midgut relative mRNA levels. **A**- *DefA* mRNA levels in anterior midgut. **B**- *DefA* mRNA levels in posterior midgut. **C**- *DefB* mRNA levels in anterior midgut. **D**- *DefB* mRNA levels in posterior midgut. **E**- *DefC* mRNA levels in anterior midgut. **F**- *DefC* mRNA levels in posterior midgut. Treatments: black column - insects fed with blood alone (control); grid column - insects fed with blood plus *E. coli*; striped column - insects fed with blood plus *S. aureus*. Error bars represent SD of three independent experiments. Asterisks relate to significant differences (*p < 0.05, **p < 0.01, ***p < 0.001) obtained after data statistical analyses using one way ANOVA and unpaired t Test.

In the anterior midgut at 1 DAF, oral infection with either *E. coli* or *S. aureus* increased mRNA levels of some AMPs in comparison with the control insects fed blood alone (Figure 5). In this tissue, *DefA* and *DefB* transcript abundance was upregulated by *S. aureus* infection (Figure 5A; p < 0.001 and 5C; p < 0.01) while *DefC* was upregulated by *E. coli* (Figure 5E; p < 0.001). In contrast, in the posterior midgut, at 1 or 7 DAF, bacterial feeding did not significantly increase the expression of *DefA* and *DefB* encoding genes (Figure 5B and 5D), although an increased expression of *DefC* 1 DAF occurred after *S. aureus* infection. (Figure 5F; p < 0.001). The transcript abundances of *DefA*, *DefB* and *DefC* were similar or even significantly lower in insects infected by either *E. coli* or S*. aureus*, in both the anterior and posterior midguts at 7 DAF when compared with control insects (Figure 5).

**Figure 6:**
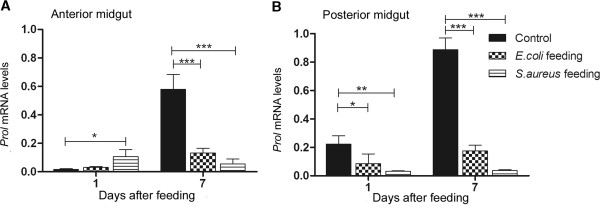
**Relative transcript abundance of prolixicin encoding mRNA in *Rhodnius prolixus *midgut wall after bacterial feeding.** Anterior and posterior midgut samples collected 1 and 7 days after feeding. **A**: anterior midgut relative mRNA levels. **B**: posterior midgut relative mRNA levels. Treatments: black column - insects fed with blood alone (control); grid column - insects fed with blood plus *E. coli*; striped column - insects fed with blood plus *S. aureus*. Error bars represent SD of three independent experiments. Asterisks relate to significant differences (*p < 0.05, **p < 0.01, ***p < 0.001) obtained after data analyses using one way ANOVA and unpaired t tests.

Concerning the *Prol* expression in both midgut tissues, only infection with *S. aureus* caused a significant increase in this AMP expression in the anterior midgut 1 DAF, when compared with control insects (Figure 6A; p < 0.05). In all other cases, *Prol* was significantly downregulated (Figure 6), especially at 7 DAF with bacteria (Figure 6A and 6B; p < 0.001).

**Figure 7:**
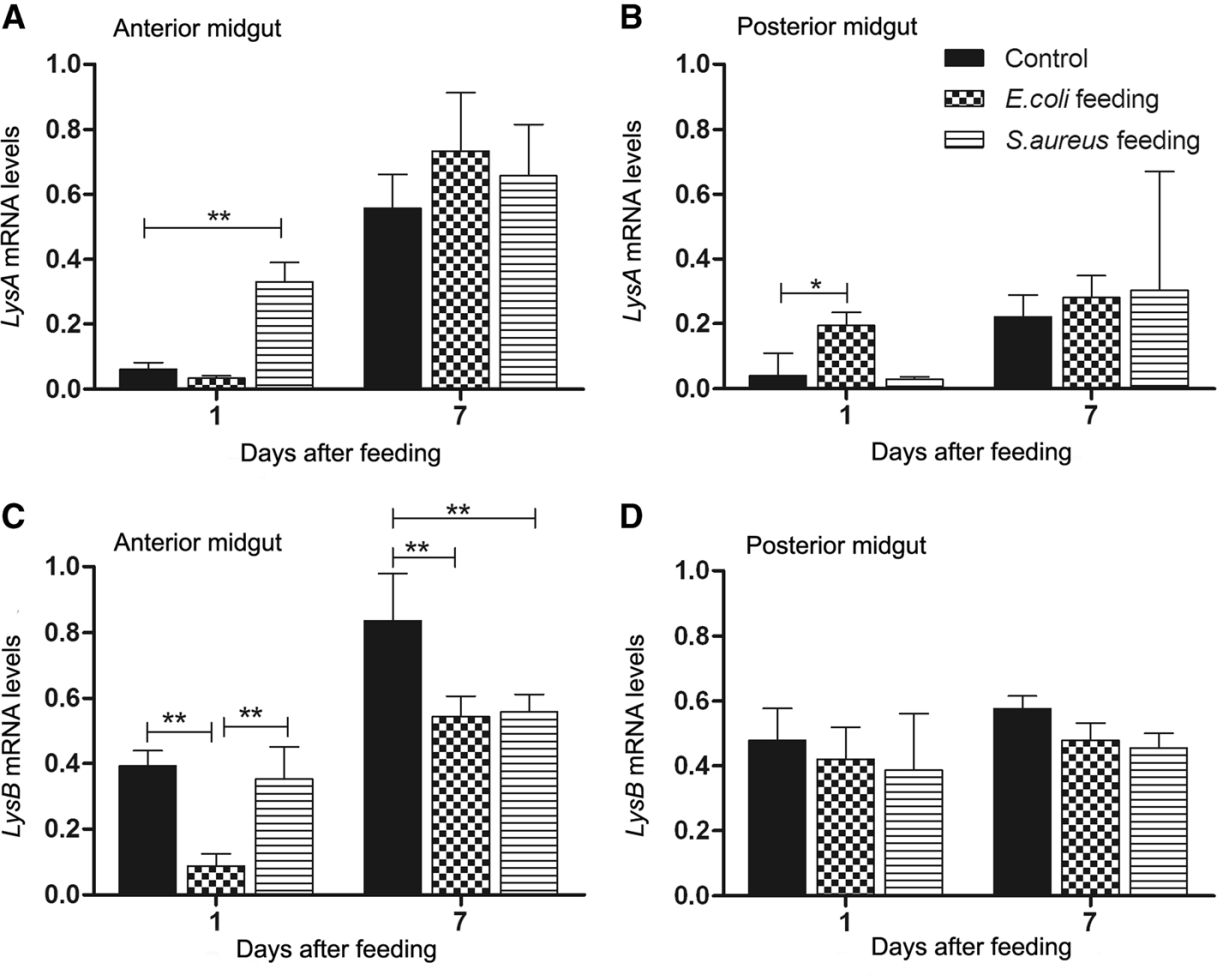
**Relative transcript abundance of lysozymes encoding mRNA in *Rhodnius prolixus *midgut after bacterial feeding.** Anterior and posterior midgut samples collected 1 and 7 days after blood meal. A, C: anterior midgut relative mRNA levels. B, D: posterior midgut relative mRNA levels. **A**- *LysA* mRNA levels in anterior midgut. **B**- *LysA* mRNA levels in posterior midgut. **C**- *LysB* mRNA levels in anterior midgut. **D**- *LysB* mRNA levels in posterior midgut. Treatments: black column - insects fed with blood; grid column - insects fed with blood plus *E. coli*; striped column - insects fed with blood plus *S. aureus*. Error bars represent SD of three independent experiments. Asterisks relate to significant differences (*p < 0.05, **p < 0.01, ***p < 0.001) obtained after data statistical analyses using one way ANOVA and unpaired t Test.

Results with lysozyme at 1 DAF showed that *LysA* was significantly upregulated in the anterior midgut after *S. aureus* infection (p < 0.01) while *LysB* was significantly downregulated after *E. coli* infection (Figure 7A and 7C; p < 0.01). In contrast, in the posterior midgut 1 DAF with *E. coli* resulted in a significant increase in *LysA* transcript abundance compared to control (Figure 7B; p < 0.05). At 7 DAF, the abundance of *LysA* and *LysB* transcripts in insects infected with either bacterial species showed similar results to control insects in both tissues (Figure 7A and 7B) except for a significant decrease in *LysB,* abundance in anterior midgut tissues of *E. coli* and *S. aureus-*infected insects (p < 0.01) when compared with controls (Figure 7C).

## Discussion

Antimicrobial peptides (AMPs) are an important part of the immune response in insects, particularly in the midgut lumen of vector species that transmit parasites during blood feeding. Furthermore, insect vectors possess gut microbiota composed of mutualistic and pathogenic bacteria [14] which are modulated by the AMPs to maintain the gut homeostasis [21]. In the present study, the results showed that oral infection with Gram-positive and Gram-negative bacteria differentially altered the antimicrobial activity and AMP expression patterns in the insect’s midgut.

The AMPs detected in the gut of *R. prolixus* in the present study included transcripts for lysozyme A (*LysA*), lysozyme B (*LysB*), prolixicin (*Prol*), defensins A (*DefA*), B (*DefB*) and C (*DefC*), although probably more AMPs await discovery in *Rhodnius*. In a recent paper by Ribeiro *et al*. [46] eight defensin and five lysozyme encoding sequences were reported. From the eight reported defensin transcripts, four were identified as *DefC*, three as *DefA* and one as a truncated *def4* of *T. brasiliensis* and no *R. prolixus DefB* was identified. However, *T. brasiliensis Def4* and *R. prolixus DefA* are highly similar and are probably orthologs. Defensins are highly conserved and therefore incomplete sequences might match with the wrong sequences deposited in the GenBank. Our study analysed all full so far identified defensin genes including *DefB* which was not found by Ribeiro *et al.*[46]. In the case of lysozyme Ribeiro *et al.*[46] identified three of the five transcripts as lysozyme 1 (syn. of *R. prolixus* lysozyme A), which was included in our study. Our results also report the presence of prolixicin, another antibacterial peptide, which was not detected by Ribeiro *et al*. [46].

Both midgut compartments were analysed for antibacterial activity, since it has been shown that each midgut compartment has a highly specific environment and physiological function [47,48]. The anterior midgut of triatomines, which has a neutral-basic pH, is where the blood meal is stored and the majority of bacterial symbionts reside. In contrast, the posterior midgut, with an acidic pH, is where protein digestion mainly occurs [45,49]. The ingested blood meal stored in the anterior midgut induces within days the transcription of AMPs and lysozymes. Concomitantly, we observed that the antibacterial activity *in vitro* was very high against both *E. coli* and *S. aureus*, reaching the highest level at 7 days after a blood meal, which may be explained by an increase of these peptides.

The results with *R. prolixus* fed with blood without microorganisms showed higher levels of mRNA encoding AMPs in the anterior than in the posterior midgut of *R. prolixus*. The anterior midgut contents also recorded a higher antibacterial activity than posterior midgut, in agreement with previous results [50]. However, results obtained by antibacterial assays on extracts from different gut regions, as in Figure 1, should be treated with caution since the anterior midgut contents are largely composed of the residual blood meal and therefore it is difficult to evaluate how much to dilute this sample to be “equivalent” to the anterior or posterior midgut walls. Thus, the protein concentration in the anterior midgut contents was 18 times higher than in the posterior midgut, and assuming that part of this protein can be related to the amount of AMPs present, then this may explain the stronger antibacterial activity detected in the anterior midgut tissues. The results showing that the most abundant AMP encoding mRNAs were present in the anterior midgut, namely, *LysA*, *LysB, DefC*, seem to confirm the elevated antimicrobial activity recorded. In addition, the inhibition of antibacterial activity observed in the anterior midgut content treated with trypsin or incubated at 100°C indicate that AMPs and lysozymes are the molecules involved [40]. Nevertheless, it is unlikely that all the antimicrobial activity recorded derived solely from these peptides since reactive oxygen (ROS) and nitrogen species (RNS) have been detected previously [21,41,50].

Regarding the results of insects fed with blood plus bacteria, this altered the pattern of antibacterial activity *in vitro* in the midgut. Feeding the insect with *S. aureus* increased the antibacterial activity against *S. aureus* in the anterior midgut and feeding the insect with *E. coli* enhanced the activity against *S. aureus* in the posterior midgut. These findings suggest that *R. prolixus* modulates antibacterial activity upon ingestion of bacteria with patterns that are distinct and dependent upon the species of bacteria present.

The results with mRNA expression showed that the *E. coli* infected insects 1 DAF expressed more *LysA* in the posterior midgut than naive insects, which may contribute to the increase in antibacterial activity against *S. aureus* observed in the posterior midgut at 7 DAF (compare Figure 4D with Figure 7B). In addition, *S. aureus* infection enhanced the anterior midgut activity against *S. aureus in vitro* and *S. aureus* infected insects showed a significantly higher *DefA*, *DefB* and *LysA* transcription abundance 1 DAF (compare Figure 4B with Figure 5A, C and Figure 7A). The significant increase in the abundance of these AMP mRNAs observed at 1 DAF may reflect an increase in antibacterial activity through to 7 DAF. This seems likely as the antibacterial activity recorded in the blood-fed controls continues to increase from 1, to 5 to 7 DAF. The enhanced *DefA*, *DefB* and *LysA* levels may explain the increase of antibacterial activity against *S. aureus* in the anterior midgut, since the respective peptides possess activity mainly against Gram-positive bacteria. Possibly other unknown *R. prolixus* antimicrobial peptides could also be responsible for the antibacterial activities observed.

In *R. prolixus,* lysozymes are involved in the digestion of polysaccharides of the symbiont *Rhodococcus rhodnii*[51]. These lysozymes may also play a role in the insect immune response [52,53]. *LysA* and *LysB* seem to have different roles in different compartments of the gut. *LysA* is expressed predominantly in the midgut and *LysB* in the fat body [31]. The rapid increase in *LysB* mRNA levels and, to a lesser extent *LysA,* in both tissues suggest a role in *R. prolixus* digestion, although a function in response to bacterial multiplication in the gut following a blood meal is also likely, as observed in other triatomines like *Triatoma infestans* and *Triatoma brasiliensis*[31,54,55]. Although phylogenetic analyses indicate that *R. prolixus LysA* groups with lysozymes that play a digestive role in other triatomine bugs [31,54,55], our results showed that *LysA* was strongly induced after *S. aureus* feeding and it was also possible to detect a slight increase of *LysA* after infection with *E. coli*, indicating also an immunological role for this lysozyme. Previous results with *Lutzomyia longipalpis* and *Galleria mellonella* have shown that there is a synergistic effect between lysozymes and other AMPs which enhances immune responses against both Gram-positive and Gram-negative bacteria [56,57]. In *R. prolixus*, synergistic effects between AMPs and lysozymes might occur as well.

Insect defensins have major activities against Gram-positive bacteria [58], but also can act against Gram-negative forms [59]. A previous study – based on structural properties – showed a high similarity between *R. prolixus DefA* and *DefB* while *DefC* differed significantly, forming two distinct groups after a phylogenetic analysis [60]. These authors suggested that the various defensins have different functions. In the present study, the analysis of transcript abundance also showed significant differences between the three *R. prolixus* defensins. In insects fed with *S. aureus* both *DefA* and *DefB* were significantly upregulated while *DefC* abundance increased only after *E. coli* infection. In unfed bugs and bugs fed solely on blood, *DefC* was the most abundant defensin transcript in the anterior midgut. The fact that in starved insects only *DefC* transcripts are abundant indicates a role of *DefC* in symbiont control whereas the upregulation of *DefA/B* after infections with unfamiliar microbes suggests a probable function of these gene products in the control of bacterial invasion. Regarding previous work, a common bacterial species found in *R. prolixus* gut was a Gram-negative bacterium, *S. marcescens*[61] and together with our findings about *DefC,* this reinforces the idea that this defensin may also play a role in the regulation of Gram-negative bacteria. As observed previously in *L. longipalpis*, high levels of defensin could be explained by microbiota control before adult emergence [62]. In *R. prolixus* fifth-instar nymphs moult to adults following a blood meal so that the high *DefC* levels could also be related to metamorphosis. Insect metamorphosis, however, is characterised not only by the need to control microbial expansion but also to mediate developmental processes and defensins have been shown to play dual roles both in immunity and development [63].

The blood meal also induces an increase of prolixicin encoding mRNA in the posterior midgut. Prolixicin, recently isolated from *R. prolixus* midgut and fat body, is a glycine-containing peptide, which is upregulated in fat body after bacterial or *Trypanosoma cruzi* haemocoel injection. The purified protein has a strong action against Gram-negative bacteria [37]. However, in the present work, prolixicin encoding gene was down-regulated in the midgut after feeding *R. prolixus* with blood containing *E. coli*. Further analyses will be necessary to clarify whether or not prolixicin is related to other microorganisms in the midgut, like e.g. different bacterial species, fungi or viruses.

In *R. prolixus*, the microbiota grows exponentially until eight days after blood feeding and thereafter decreases [50,64]. This might explain why a higher expression of peptides occurs on 7 DAF. Since AMPs may have a central role in the control of bacterial populations in the midgut. This would also explain the higher expression of the AMP encoding mRNAs and AMPs in the anterior midgut than in the posterior midgut since the anterior midgut, including the lumen, is the site of the bacterial bloom resulting from the blood meal. The anterior midgut may be a more suitable environment for lysozymes while prolixicin would control bacterial expansion in the posterior midgut.

The activation of immune responses in insects is regulated mainly by two intracellular pathways, the Toll and the IMD pathways [65,66], that control the expression of most genes encoding the AMPs. Gram-positive bacterial infection activate the Toll pathway while Gram-negative bacteria infection induces the IMD pathway [67]. In the present work, different types of bacterial infection induced the expression of different types of AMPs in insect’s midgut. Thus, AMP encoding genes induced by *S. aureus* (*DefA*, *DefB*, *Prol*) and *E. coli* (*DefC*) infections could be under different induction pathways.

## Conclusion

Studies of the activation of immune responses in the gut become more relevant than those responses triggered by artificial inoculation in the body cavity of the insect, since these events occur less frequently in nature [67]. Insects and other animals live in a complex relationship with microorganisms [68] and the study of transcriptional control of AMPs can extend the understanding of how insects manage microbiota interactions and are still able to mount an efficient immune response against possible ingested pathogens.

## Competing interests

The authors declare that they have no competing interests.

## Authors’ contributions

CSV, PJW, DPC, PA, NAR, ESG and CBM designed the study protocols and drafted the manuscript; CSV, DPM and DPC carried out the antibacterial activity experiments; CSV, PJW, DPM and DPC performed the molecular experiments. All authors analyzed the data, revised the article, approved the version to be published and are the guarantors of the paper.

## Supplementary Material

Additional file 1 **Antibacterial activity of anterior midgut contents and posterior midgut of *Rhodnius prolixus *** **(7 DAF) tested against *Escherichia coli *** **and *Staphylococcus aureus*.** The activity was measured as colony forming units (CFU/ml) after 19 hours of incubation. Values represent the means ± SD of 9 pools using 3 insects (n = 27) in triplicate wells. Click here for file

Additional file 2 **Antibacterial activity from *R. prolixus *** **anterior midgut fed on normal blood and washed erythrocytes with inactivated plasma.** Antibacterial activity was measured by turbidometric assay (TB) (OD_550_ nm) with readings from hour 0 to hour 20 of incubation in plate assay. **A:** Activity against *E. coli*. **B** – Activity against *S. aureus.* Treatments: ■ bacteria incubated with content of anterior midgut from insects fed on blood; ● bacteria incubated with anterior midgut from insects fed on inactivated plasma (IP) blood; ♦ bacteria incubated with posterior midgut from insects fed on blood. ▲ bacteria incubated with posterior midgut from insects fed on erythrocytes with inactivated plasma (IP) blood. Values represent the means ± SD of 9 pools using 3 insects each (n = 27) in triplicate wells. Statistical analysis was carried out using two way ANOVA. Click here for file

Additional file 3 **Antibacterial activity of anterior midgut of *Rhodnius prolixus *** **at 7 days after blood meal.** Antibacterial activity detected by turbidometric assay (TB) (OD_550_ nm) after 19 hours of incubation of anterior midgut samples with different bacteria. **A** – Antibacterial activity against *Escherichia coli*. **B** – Antibacterial activity against *Staphylococcus aureus*. Treatments: Black column - incubated with untreated anterior midgut; grid column - *bacteria* incubated with anterior midgut treated 24 hours with trypsin; striped column - *bacteria* incubated with anterior midgut heated at 100°C; Values represent the means ± SD of three replicates. Asterisks relates to significant differences (*p < 0.05, **p < 0.01, ***p < 0.001) obtained after data statistical analyses in comparison to control using one way ANOVA and Mann Whitney test. Click here for file
