# Supplementary material for: Humoral responses in Rhodnius prolixus: bacterial feeding induces differential patterns of antibacterial activity and enhances mRNA levels of antimicrobial peptides in the midgut
Source: Parasit Vectors. 2014 May 20;7:232. doi: 10.1186/1756-3305-7-232 (PMC4032158; doi:10.1186/1756-3305-7-232)
Supplement: Additional file 1 — Antibacterial activity of anterior midgut contents and posterior midgut of Rhodnius prolixus (7 DAF) tested against Escherichia coli and Staphylococcus aureus . The activity was measured as colony forming units (CFU/ml) after 19 hours of incubation. Values represent the means ± SD of 9 pools using 3 insects (n = 27) in triplicate wells. [file 1756-3305-7-232-S1.docx]

**Additional file 1:** Antibacterial activity of anterior midgut contents and posterior midgut of *Rhodnius prolixus* (7 DAF) tested against *Escherichia coli* and *Staphylococcus aureus*. The activity was measured as colony forming units (CFU/ml) after 19 hours of incubation. Values represent the means ± SD of three replicates.

| **Insect samples** | ***Escherichia coli Staphylococcus aureus*** | | | |
| --- | --- | --- | --- | --- |
|  | **Incubation time (h) (CFU/ml)** | | | |
|  | **0h** | **19h** | **0h** | **19h** |
| **Control** | 8.3 x 10^3^ + 2.8 | 1.3 x 10^10^ + 0.6 | 4.1 x 10^3^ + 2.8 | 6.2 x 10^11^ + 2.5 |
| **Anterior midgut** | 6.1 x 10^3^ + 2.2 | 0* | 2.4 x 10^3^ + 0.8 | 0* |
| **Posterior midgut** | 4.9 x 10^3^ + 2.3 | 2.3 x 10^10^ + 0.6 | 3.5 x 10^3^ + 1.9 | 1.8 x 10^9^ + 2.1 |

*Since 2 hours of incubation of both bacteria with anterior midgut, no bacteria was detected on agar plates.
