# Supplementary figures and images for: Humoral responses in Rhodnius prolixus: bacterial feeding induces differential patterns of antibacterial activity and enhances mRNA levels of antimicrobial peptides in the midgut
Source: Parasit Vectors. 2014 May 20;7:232. doi: 10.1186/1756-3305-7-232 (PMC4032158; doi:10.1186/1756-3305-7-232)

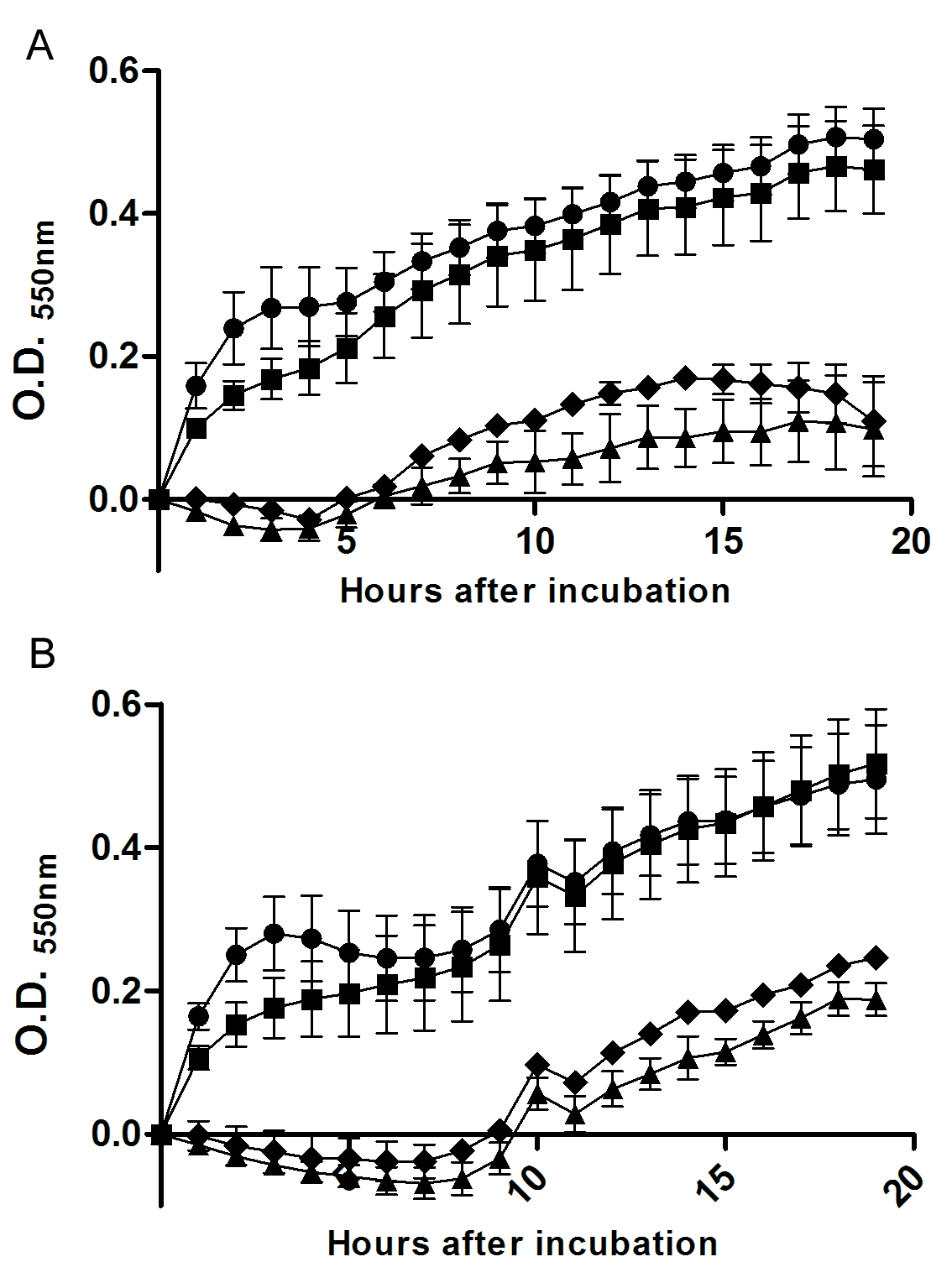

Supplement: Additional file 2 — Antibacterial activity from R. prolixus anterior midgut fed on normal blood and washed erythrocytes with inactivated plasma. Antibacterial activity was measured by turbidometric assay (TB) (OD550 nm) with readings from hour 0 to hour 20 of incubation in plate assay. A: Activity against E. coli. B – Activity against S. aureus. Treatments: ■ bacteria incubated with content of anterior midgut from insects fed on blood; ● bacteria incubated with anterior midgut from insects fed on inactivated plasma (IP) blood; ♦ bacteria incubated with posterior midgut from insects fed on blood. ▲ bacteria incubated with posterior midgut from insects fed on erythrocytes with inactivated plasma (IP) blood. Values represent the means ± SD of 9 pools using 3 insects each (n = 27) in triplicate wells. Statistical analysis was carried out using two way ANOVA. [file 1756-3305-7-232-S2.tiff]

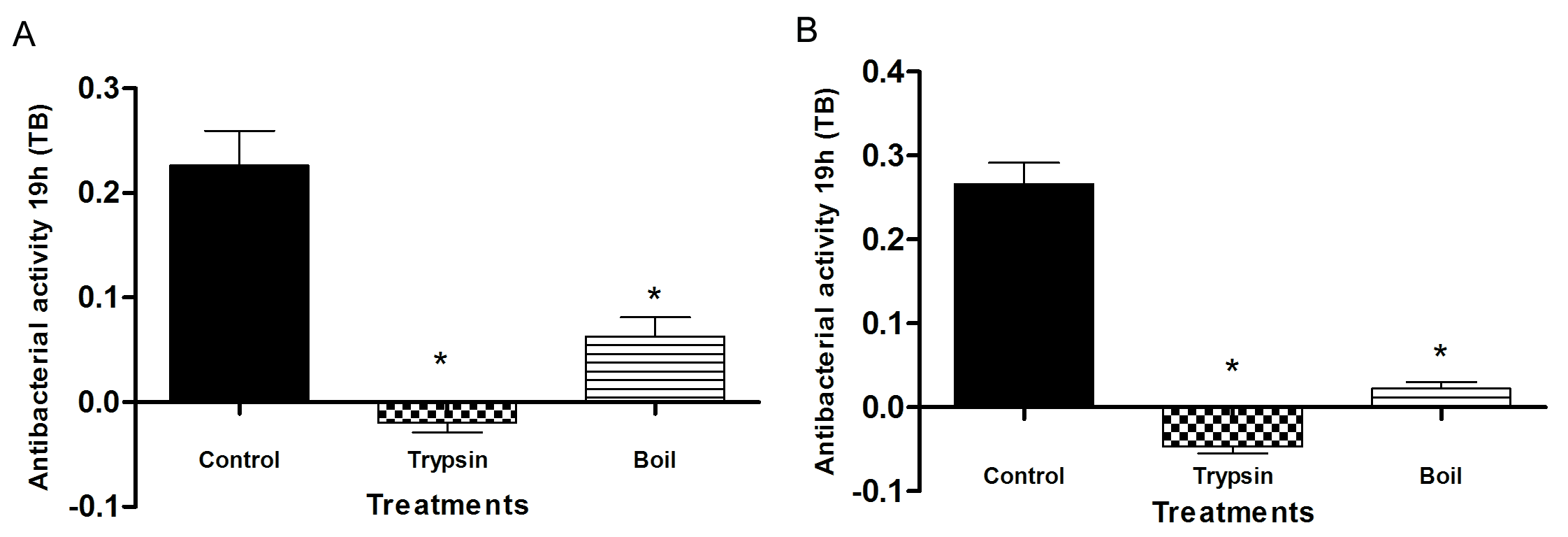

Supplement: Additional file 3 — Antibacterial activity of anterior midgut of Rhodnius prolixus at 7 days after blood meal. Antibacterial activity detected by turbidometric assay (TB) (OD550 nm) after 19 hours of incubation of anterior midgut samples with different bacteria. A – Antibacterial activity against Escherichia coli. B – Antibacterial activity against Staphylococcus aureus. Treatments: Black column - incubated with untreated anterior midgut; grid column - bacteria incubated with anterior midgut treated 24 hours with trypsin; striped column - bacteria incubated with anterior midgut heated at 100°C; Values represent the means ± SD of three replicates. Asterisks relates to significant differences (*p < 0.05, **p < 0.01, ***p < 0.001) obtained after data statistical analyses in comparison to control using one way ANOVA and Mann Whitney test. [file 1756-3305-7-232-S3.tiff]
